# Mitochondrial DNA Variation and Disease Susceptibility in Primary Open-Angle Glaucoma

**DOI:** 10.1167/iovs.18-25085

**Published:** 2018-09

**Authors:** Larry N. Singh, Jonathan G. Crowston, M. Isabel G. Lopez Sanchez, Nicole J. Van Bergen, Lisa S. Kearns, Alex W. Hewitt, Seyhan Yazar, David A. Mackey, Douglas C. Wallace, Ian A. Trounce

**Affiliations:** 1 Center for Mitochondrial and Epigenomic Medicine, Children's Hospital of Philadelphia Research Institute, University of Pennsylvania, Philadelphia, United States; 2 Center for Eye Research Australia, Ophthalmology, University of Melbourne Department of Surgery, Melbourne, Australia; 3 Menzies Research Institute Tasmania, School of Medicine, University of Tasmania, Hobart, Australia; 4 Lions Eye Institute, University of Western Australia, Centre for Ophthalmology and Visual Science, Perth, Australia; 5 MRC Human Genetics Unit, Institute of Genetics and Molecular Medicine, University of Edinburgh, Edinburgh, United Kingdom; 6 Department of Pathology and Laboratory Medicine, University of Pennsylvania, Philadelphia, United States

**Keywords:** glaucoma, mitochondrial DNA (mtDNA), haplogroup

## Abstract

**Purpose:**

To determine whether mitochondrial DNA haplogroups or rare variants associate with primary open-angle glaucoma in subjects of European descent.

**Methods:**

A case–control comparison of age- and sex-matched cohorts of 90 primary open-angle glaucoma patients and 95 population controls. Full mitochondrial DNA sequences from peripheral blood were generated by next-generation sequencing and compared to the revised Cambridge Reference Sequence to define mitochondrial haplogroups and variants.

**Results:**

Most subjects were of the major European haplogroups H, J, K, U, and T. Logistic regression analysis showed haplogroup U to be significantly underrepresented in male primary open-angle glaucoma subjects (odds ratio 0.25; 95% confidence interval [CI] 0.09–0.67; *P* = 0.007; Bonferroni multiple testing *P* = 0.022). Variants in the mitochondrial DNA gene *MT-ND2* were overrepresented in the control group (*P* = 0.005; Bonferroni multiple testing correction *P* = 0.015).

**Conclusions:**

Mitochondrial DNA ancestral lineages modulate the risk for primary open-angle glaucoma in populations of European descent. Haplogroup U and rare variants in the mitochondrial DNA-encoded *MT-ND2* gene may be protective against primary open-angle glaucoma. Larger studies are warranted to explore haplogroup associations with disease risk in different ethnic groups and define biomarkers of primary open-angle glaucoma endophenotypes to target therapeutic strategies.

Primary open-angle glaucoma (POAG) is a heterogenous disease grouping associated with selective loss of retinal ganglion cells and is a leading cause of blindness worldwide. POAG is often associated with increased intraocular pressure (IOP), but many cases progress despite IOP being within normal ranges.^1^ The specificity of retinal ganglion cell loss in POAG resembles mitochondrial optic neuropathies, which are rare diseases caused by genetic defects in either nuclear-encoded mitochondrial genes or mitochondrial DNA (mtDNA) genes.^2^ In both these disease groupings retinal ganglion cells are lost in the absence of other central nervous system pathology. The pattern of axonal loss in the optic nerve can be distinct, however, with preferential loss of smaller-caliber parvocellular axons in mitochondrial optic neuropathies leading to central scotomas, while in POAG larger-caliber magnocellular fibers tend to be preferentially affected leading to more arcuate field defects.^3,4^ POAG fiber loss can vary from this classic pattern, with many cases involving central field defects,^5^ possibly reflecting the heterogenous nature of the disease group. The broad similarities of optic nerve degeneration in mitochondrial optic neuropathies and POAG have been long noted, driving interest in studying mitochondrial function and mtDNA variation in POAG.^6–8^ Studies using peripheral blood-derived lymphoblast cell lines have reported evidence of oxidative phosphorylation (OXPHOS) complex I defects in POAG.^9,10^ Further exploration of mitochondrial function and genetics in POAG may lead to markers of a subtype with a mitochondrial etiology.

An increased incidence of POAG among patients' first-degree relatives is well established; relatives of POAG patients have a 22% risk of developing POAG at some point in their lives, whereas the risk for the relatives of controls is 2% to 3%.^11^ This risk has been shown to be two to five times greater on the maternal side of families.^12^ However, only two genes, myocilin (*MYOC*) and optineurin (*OPTN*), have been established to cause familial POAG with high penetrance. Myocilin mutations account for 2% to 4% of POAG and are associated with high IOP, while optineurin mutations may account for 1% of POAG and 2% of normal-tension glaucoma.^11^ MtDNA variation has been hypothesized to be involved in POAG risk, with studies suggesting increased levels of rare mtDNA variants in POAG cases.^13,14^ While interesting findings are emerging, systematic studies to date have not always considered the population structure of mtDNA ancestry in their analyses. MtDNA is exclusively maternally inherited, and several ancient single nucleotide variants (SNVs) are inherited as a group, termed a haplogroup. MtDNA haplogroups represent human populations having the same ancestral origins and migration patterns and have been associated with disease risk.^15,16^ Here, we investigated the potential association between mtDNA haplogroups and rare mtDNA variants in POAG in a clinically defined cohort of patients and population controls of European descent.

## Methods

### Participants

Inclusion criteria for the study were as follows: evidence of glaucomatous optic neuropathy identified by loss of the neuroretinal rim and retinal nerve fiber layer loss, and corresponding visual field defect and open angles on gonioscopy. All patients and population controls underwent a detailed medical history and slit-lamp biomicroscopy as well as blood sampling for lymphocyte DNA isolation. The IOP was measured by Goldmann applanation tonometry and all assessments were performed at 3 PM ± 1 hour. All patients were receiving treatment for POAG at the time of recruitment. Subjects with congenital angle-closure glaucoma, trauma-associated secondary glaucoma, or neovascular glaucoma were excluded. The study adhered to the tenets of the Declaration of Helsinki; institutional human ethics committee (Royal Victorian Eye and Ear Hospital) approval was granted, and written informed consent was acquired from all patients.

### Data Acquisition and Sequencing

MtDNA sequencing was undertaken by the Australian Genome Research Facility using the Illumina HiSeq platform (Illumina, Inc., San Diego, CA, USA). From 100 ng total DNA per sample, entire mtDNAs were amplified in two overlapping fragments with PCR primers known to avoid nuclear mtDNA pseudogene (NUMT) sequences.^17^ The PCR fragments were combined in equal ratios, and PCR amplicons enzymatically fragmented using NEBNext dsDNA Fragmentase (New England Biolabs, Ipswich, MA, USA). Illumina-compatible NEXTflex PCRfree 48-plex barcode oligonucleotide adapters (Bio Scientific, Sydney, New South Wales, Australia) were then ligated. Following size selection on a 2% agarose gel, the cluster formation and sequencing by synthesis were performed using an Illumina HiSeq2000 instrument, 101-bp (base pair) paired-end reads, and V3 reagents according to the manufacturer's protocols (Illumina, Inc.). Average read depth achieved was 10,000.

Since mtDNA is exclusively maternally inherited, there is no recombination and many mtDNA variants are in high linkage disequilibrium (LD), that is, occur on the same DNA molecule. Indeed, this fact leads to the presence of mitochondrial haplogroups. The majority of mtDNA-encoded genes are involved in the electron transport chain, and hence it is likely that these mtDNA variants are in high LD to allow coevolution of the various components of the electron transport chain to adapt to different environments and conditions. Due to this high LD in mtDNA, to avoid errors in statistical computations, we computed representative tag SNVs for variant association analysis with POAG, and all single variant analyses were computed using these tag SNVs.

### Sequencing Analysis

Paired-end sequence reads in fastq files were aligned with bwa mem version 0.7.15^18^ using default alignments to the revised Cambridge Reference Sequence (rCRS - NC_012920.1). Duplicate reads were identified using Picard tools version 1.130 (http://broadinstitute.github.io/picard; in the public domain). Variants were called using freebayes, a haplotype-based variant detector version 1.1.0^19^ with arguments “-p 1 –C 2 –q 10.” Using homoplasmic mtDNA variant calls only, haplogroups were then inferred using Haplogrep software version 2.1.0.^20^ To avoid errors in statistical computations, we computed representative tag SNVs for variant association analysis with POAG, and all single variant analyses were computed using these tag SNVs. Tag SNVs were computed from 30,589 full mtDNA sequences from mitomap (http://www.mitomap.org; in the public domain), using PLINK 2.0 (http://www.cog-genomics.org/plink/2.0/; in the public domain), and Lewontin's D' statistic as a measure of LD.

### Statistical Analysis

All statistical analyses were performed using R version 3.2.1 (R Foundation, Vienna, Austria). All *P* values were reported after Bonferroni multiple testing correction, where applicable. Burden tests were performed with the adaptive sum of powered scores (aSPU) tests,^21^ using 20,000 permutations.

## Results

### Male Individuals in Mitochondrial Haplogroup U Have a Lower Risk for POAG

Mitochondrial haplogroups for 90 POAG patients and 95 population controls in this study were inferred from homoplasmic mtDNA. The haplogroup distribution we obtained showed a predominantly Western European maternal ancestry of our study population, which was closely matched between cases and controls. This reflects the older demographic sampled in Australia. Due to the small numbers of individuals in some haplogroups, we combined haplogroups according to the mtDNA phylogenetic tree (phylotree build 17).^22^ Demographic and haplogroup information for this study cohort is included in Supplementary Table S1. To determine if the haplogroup of an individual is a significant predictor for POAG, we used logistic regression analysis with the POAG status of the individual as the response variable, while adjusting for age. We defined haplogroup R_U_ as the European root macro-haplogroup R (including haplogroups B, F, H, HV, J, R, T, V) minus haplogroup U. We disregarded haplogroup L because there was only one person from this group in our study. We found that men in haplogroup U were at approximately four times lower risk for POAG compared to men in haplogroup R_U_ (Table 1). In our study, there was no association between POAG risk and mtDNA haplogroup in the cases when both sexes were combined (Supplementary Table S2).

**Table 1:**
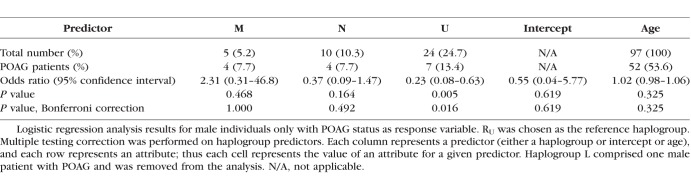
Men in Haplogroup U Are Underrepresented in POAG

### Rare mtDNA SNVs Are Associated With POAG

To avoid private mutations, a rare mtDNA variant was defined as a variant that was not common (see below) and was present in at least three people. There were 133 unique mtDNA rare variants detected in the data set. We aggregated rare variants within mtDNA genes to increase statistical power and applied the genetic burden aSPU tests implemented in the aSPU R package.^21^ We also restricted analysis to genes having at least 25 nonreference variants, which resulted in two genes and the noncoding regulatory D-Loop region. We found that individuals having rare variants in the *MT-ND2* gene were at significantly lower risk for POAG than individuals without these variants (Table 2).

**Table 2:**
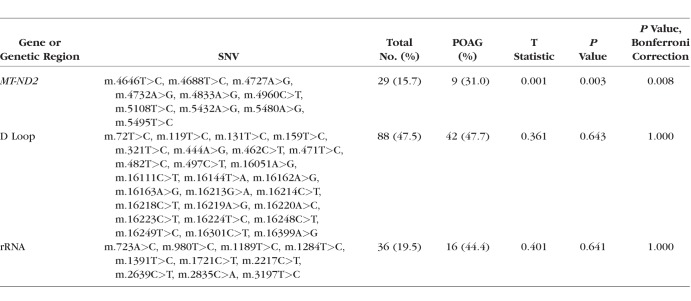
aSPU Analysis of Rare mtDNA Variants Detected

We defined common mtDNA SNVs as those variants having a minor allele frequency of at least 15%, which yielded nine unique common variants (Supplementary Table S3). To determine if common mtDNA SNVs were associated with POAG, we performed logistic regression analysis with disease status as the response variable and age, sex, and common variants as covariates. None of the common mtDNA variants were significantly associated with POAG after multiple testing correction at the 0.05 level.

## Discussion

Several studies have investigated mtDNA variation in POAG, but few have used a phylogenetic approach to consider variants either inherited as a group (haplogroups) or the genetic burden of multiple rare mtDNA variants, as we report here. Some earlier reports have led to spurious conclusions due to a lack of consideration of mitochondrial haplogroups. Abu-Amero et al.^23^ sequenced the entire mtDNA of 27 Arabic POAG patients, finding 34 nonsynonymous sequence variants not detected in 159 population controls. However, when the same group later considered haplogroups in an expanded population sample, they found that African haplogroups were enriched in their case group, accounting for the increased levels of variants in their POAG cohort.^24^

Other studies have not considered mtDNA haplogroups. Banerjee et al.^13^ sequenced entire mtDNAs from 101 Indian POAG patients and 71 controls. The authors also found significantly increased nonsynonymous SNVs in mtDNA of POAG patients, particularly in complex I genes, and suggested the *MT*-*ND5* gene as a mutation “hot spot” in POAG. Most of their patients and controls belonged to haplogroup M, which predominates in the Indian subcontinent. Yet their patient group included 18 individuals of the European root haplogroup R while their control group included only 5 haplogroup R individuals. Such disparities lead to apparent differences in levels of variants seen. In their list of rare variants found only in POAG cases, the variant m.5178C>A is a defining SNV for haplogroup D; the variant m.9055G>A is a defining SNV for haplogroup K; the variant m.14000T>A is commonly associated with haplogroup L1c (Table 3 in Banerjee et al.^13^). Thus three out of four “rare variants” that were recorded multiple times are explained by rare haplogroups in their population. Another study of 32 POAG subject mtDNAs found higher levels of rare variants in cases compared with controls.^14^ Half of the cases in this study were from the United Kingdom and half from India. The authors state that 110 “ethnically matched” controls were also sequenced, but no mtDNA haplogroups were reported for their cases or controls. Their table of rare variants also shows haplogroup marker SNVs. The variant m.1453A>G (two patients) is a defining SNV of haplogroup M2; variant m.3866T>C (two patients) is found on haplogroup J; variant m.4336T>C (one patient) is found on haplogroup M5; variant m.12397A>G (two patients) is found on haplogroup M19.^14^ This finding shows the need to first consider haplogroup associations, and then sort rare variants that conflict with the patient haplogroup, as we have done. Common pitfalls in mtDNA disease association studies have been critically reviewed elsewhere.^25^

Another group has focused on African American POAG patients, initially reporting no differences in rare variant levels between cases and controls.^26^ This group then used a mtDNA haplogroup approach, finding African haplogroups L1c2, L1c2b, and L2 significantly enriched in their cases.^27^ Our analyses in a population of predominantly Western European ancestry showed some surprising associations for the relatively small sample size. We found that individuals in haplogroup U were significantly underrepresented in male POAG cases, suggesting a protective effect of these lineages for males only. A study of German glaucoma patients found haplogroup U significantly underrepresented in pseudoexfoliation glaucoma subjects, and a trend for haplogroup U being underrepresented in POAG; however, they did not investigate sex as a variable.^28^ A second finding in our study was a significantly lower frequency of rare variants in the *MT-ND2* gene in cases compared with controls, suggesting a protective effect against POAG. This finding predicts that subsets of several common variants may be required for glaucoma to progress. Complex age-related diseases such as glaucoma are likely to have environmental risk factors in addition to genetic risk factors, and different mtDNA haplogroups or rare variants may act as risk or protective “alleles” depending on the environment and cellular dependence on OXPHOS.^29^ In vitro studies have shown that human mtDNA haplogroups show differences in OXPHOS capacity.^30,31^

Studies in much larger cohorts of POAG and ophthalmologically screened population controls are warranted to further test for mtDNA influence on disease risk. Importantly, as POAG is a heterogeneous disease grouping, the role of mtDNA variation in patients stratified by normal-tension glaucoma, slightly elevated IOP, high IOP, progression rates, or visual field loss patterns requires further investigation. Our findings imply that disease risk variants in mtDNA will be uncovered in larger studies and may lead to identification of mtDNA biomarkers that define a POAG endophenotype of mitochondrial origin.

## Supplementary Material

Supplement 1
