# Supplementary material for: Mitochondrial DNA Variation and Disease Susceptibility in Primary Open-Angle Glaucoma
Source: Invest Ophthalmol Vis Sci. 2018 Sep;59(11):4598–602. doi: 10.1167/iovs.18-25085 (PMC6138263; doi:10.1167/iovs.18-25085)
Supplement: Supplement 1 [file iovs-59-11-09_s01.pdf]

Supplementary Tables

Supplementary Table S1. Case and control demographic and haplogroup distributions.

Characteristic	Control	POAG
**Total study population**	n=95	n=90
**Mean age in years** (SD)	67.04 (11.3)	69.37 (10.7)
**Sex**		
Female (%)	50 (52.6)	37 (41.1)
Male (%)	45 (47.4)	53 (58.9)
**Haplogroup**		
R_U (B, F, H, HV, J, R, T, V)	57	57
U (U1-U5, U7, K1, K2)	25	18
N (N, I, W, X)	12	8
M (M, C, G)	1	6
L	0	1

Supplementary Table S2. Logistic regression analysis results for all individuals with glaucoma status as response variable. Multiple testing correction was performed on haplogroup predictors.

**Predictor**	**M**	**N**	**U**	**Intercept**	**Age**	**Sex**
Total number (%)	7 (3.8)	20 (10.9)	43 (23.4)	N/A	184 (100)	184 (100)
POAG patients (%)	6 (6.7)	8 (9.0)	18 (20.2)	N/A	89 (100)	89 (48.4)
Odds Ratio (95% CI)	6.22 (0.99-121)	0.65 (0.24-1.73)	0.70 (0.34-1.44)	0.14 (0.01-1.06)	1.03 (1.00-1.06)	1.65 (0.90-3.03)
P value	0.099	0.396	0.336	0.070	0.096	0.106
Bonferroni-adjusted P value	0.297	1.000	1.000	0.070	0.096	0.106

Supplementary Table S3. Common mtDNA variants analyzed. No statistically significant differences were found for any of these variants. MtDNA SNV entries with multiple SNVs indicate SNVs in linkage disequilibrium.

mtDNA SNV	Total (%)	POAG patients (%)	P value	Bonferroni adjusted P value
m.73A>G	111 (60.7)	52 (46.8)	0.489	1.00
m.709G>A	32 (17.5)	19 (59.4)	0.148	1.00
m.1811A>G	25 (13.7)	10 (40.0)	0.315	1.00
m.2706A>G<br>m.7028C>T	115 (62.8)	57 (49.6)	0.591	1.00
m.3010G>A	39 (21.3)	20 (52.5)	0.571	1.00
m.4216T>C<br>m.11251A>G<br>m.15452A>C	33 (18.0)	16 (51.3)	0.923	1.00
m.11467A>G<br>m.12308A>G<br>m.12372A>G	42 (23.0)	17 (40.5)	0.245	1.00
m.11719G>A<br>m.14766C>T	108 (59.0)	52 (48.1)	0.948	1.00
m.12705C>T<br>m.16223C>T	27 (14.8)	14 (51.9)	0.676	1.00
m.14798T>C	26 (14.2)	11 (42.3)	0.580	1.00
m.16311T>C	31 (16.9)	16 (51.6)	0.720	1.00
m.16362T>C	22 (12.0)	9 (40.9)	0.639	1.00
m.16519T>C	121 (66.1)	65 (53.7)	0.054	0.76
